# A unified data representation theory for network visualization, ordering and coarse-graining

**DOI:** 10.1038/srep13786

**Published:** 2015-09-08

**Authors:** István A. Kovács, Réka Mizsei, Péter Csermely

**Affiliations:** 1 Wigner Research Centre, Institute for Solid State Physics and Optics, H-1525 Budapest, P.O.Box 49, Hungary; 2 Institute of Theoretical Physics, Szeged University, H-6720 Szeged, Hungary; 3 Center for Complex Networks Research and Department of Physics, Northeastern University, 177 Huntington Avenue, Boston, MA 02115, USA; 4 Institute of Organic Chemistry, Research Centre for Natural Sciences, Hungarian Academy of Sciences, Pusztaszeri út 59-67, H-1025 Budapest, Hungary; 5 Department of Medical Chemistry, Semmelweis University, H-1444 Budapest, P.O.Box 266, Hungary

## Abstract

Representation of large data sets became a key question of many scientific disciplines in the last decade. Several approaches for network visualization, data ordering and coarse-graining accomplished this goal. However, there was no underlying theoretical framework linking these problems. Here we show an elegant, information theoretic data representation approach as a unified solution of network visualization, data ordering and coarse-graining. The optimal representation is the hardest to distinguish from the original data matrix, measured by the relative entropy. The representation of network nodes as probability distributions provides an efficient visualization method and, in one dimension, an ordering of network nodes and edges. Coarse-grained representations of the input network enable both efficient data compression and hierarchical visualization to achieve high quality representations of larger data sets. Our unified data representation theory will help the analysis of extensive data sets, by revealing the large-scale structure of complex networks in a comprehensible form.

Complex network^1^,^2^ representations are widely used in physical, biological and social systems, and are usually given by huge data matrices. Network data size grew to the extent, which is too large for direct comprehension and requires carefully chosen representations. One option to gain insight into the structure of complex systems is to order the matrix elements to reveal the concealed patterns, such as degree-correlations^3^,^4^ or community structure^5^,^6^,^7^,^8^,^9^,^10^,^11^. Currently, there is a diversity of matrix ordering schemes of different backgrounds, such as graph theoretic methods^12^, sparse matrix techniques^13^ and spectral decomposition algorithms^14^. Coarse-graining or renormalization of networks^15^,^16^,^17^,^18^,^19^,^20^ also gained significant attention recently as an efficient tool to zoom out from the network, by averaging out short-scale details to reduce the size of the network to a tolerable extent and reveal the large-scale patterns. A variety of heuristic coarse-graining techniques – also known as multi-scale approaches – emerged, leading to significant advances of network-related optimization problems^21^,^22^ and the understanding of network structure^19^,^20^,^23^. As we discuss in the Supplementary Information in more details, coarse-graining is also closely related to some block-models useful for clustering and benchmark graph generation^24^,^25^,^26^.

The most essential tool of network comprehension is a faithful visualization of the network^27^. Preceding more elaborate quantitative studies, it is capable of yielding an intuitive, direct qualitative understanding of complex systems. Although being of a primary importance, there is no general theory for network layout, leading to a multitude of graph drawing techniques. Among these, force-directed^28^ methods are probably the most popular visualization tools, which rely on physical metaphors. Graph layout aims to produce aesthetically appealing outputs, with many subjective aims to quantify, such as minimal overlaps between not related parts (e.g. minimal edge crossings in *d* = 2), while preserving the symmetries of the network. Altogether, the field of graph drawing became a meeting point of art, physics and computer science^29^.

Since the known approaches for the above problems generally lead to computationally expensive NP-hard problems^30^, the practical implementations were necessarily restricted to advanced approximative heuristic algorithms. Moreover, there was no successful attempt to incorporate network visualization, data ordering and coarse-graining into a common theoretical framework. Since information theory provides ideal tools to quantify the hidden structure in probabilistic data^31^,^32^, its application to complex networks^25^,^26^,^33^,^34^,^35^,^36^,^37^ is a highly promising field. In this paper, our primary goal is to show an elegant, information theoretic representation theory for the unified solution of network visualization, data ordering and coarse-graining, establishing a common ground for the first time for these separated fields.

Usually, in graph theory, the complex system is at the level of abstraction, where each node is a dimensionless object, connected by lines representing their relations, given by the input data. Instead, we study the case in which both the input matrix and the approximative representation is given in the form of a probability distribution. This is the routinely considered case of edge weights reflecting the existence, frequency or strength of the interaction, such as in social and technological networks of communication, collaboration and traveling or in biological networks of interacting molecules or species. As discussed in details in the Supplementary Information, the probabilistic framework has long traditions in the theory of complex networks, including general random graph models, all Bayesian methods, community detection benchmarks^24^, block-models^25^,^26^ and graphons^38^.

The major tenet of our unified framework is that the best representation is selected by the criteria, that it is the hardest to be distinguished from the input data. In information theory this is readily obtained by minimizing the relative entropy – also known as the Kullback-Leibler divergence^39^ – as a quality function. In the following we show that the visualization, ordering and coarse-graining of networks are intimately related to each other, being organic parts of a straightforward, unified representation theory. We also show that in some special cases our unified framework becomes identical with some of the known state-of-the-art solutions for both visualization^40^,^41^,^42^ and coarse-graining^25^,^26^, obtained independently in the literature.

## Results

### General network representation theory

For simplicity, here we consider a symmetric adjacency matrix, *A*, having probabilistic entries *a*_*ij*_ ≥ 0 and we try to find the optimal representation in terms of another matrix, *B*, having the same size. For more general inputs, such as hypergraphs given by an *H* incidence matrix, see the Methods section. The intuitive idea behind our framework is that we try to find the representation which is hardest to be distinguished from the input matrix. Within the frames of information theory, there is a natural way to quantify the closeness or *quality* of the representation, given by the relative entropy. The relative entropy, 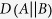, measures the extra description length, when *B* is used to encode the data described by the original matrix, *A*, expressed by


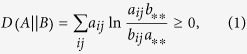


where 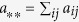 and 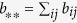 ensure the proper normalizations of the probability distributions. As a shorthand notation here and in the following an asterisk indicates in index, for which the summation was carried out, as in the cases of 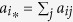 and 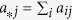. Although 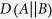 is not a metric and not symmetric in *A* and *B*, it is an appropriate and widely applied measure of statistical remoteness^43^, quantifying the distinguishability of *B* form *A*. The highest quality representation is achieved, when the relative entropy approaches 0, and our goal is to obtain a B* representation satisfying


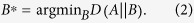


**Figure 1:**
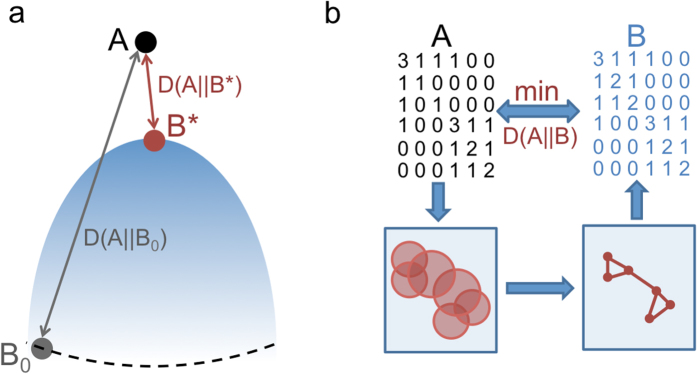
Illustration of our data representation framework. (**a**) For a given *A* input matrix, our goal is to find the closest *B* representation, measured by the 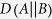 Kullback-Leibler divergence. The trivial representation, *B*_0_, is always at a finite 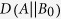 value, limiting the search space. (**b**) In the data representation example of network visualization, we assign a distribution function to each network node, from which edge weights (*B*) are calculated based on the overlaps of the distributions. The best layout is given by the representation, which minimizes the 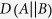 description length.

Although 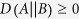 can be in principle arbitrarily large, there is always a trivial upper bound available by the uncorrelated, *product state* representation, *B*^0^, given by the matrix elements 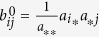. For an illustration see Fig. 1a. It follows simply from the definition of the *S*(*A*), total information content and *I*(*A*), mutual information, given in the Methods section, that 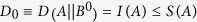. Consequently, the optimized value of 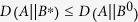 can be always normalized with *I*(*A*), or alternatively as


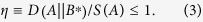


Here *η* is the ratio of the needed extra description length to the optimal description length of the system. In the following applications we use *η* to compare the optimality of the found representations. As an important property, the optimization of relative entropy is local in the sense, that the global optimum of a network comprising independent subnetworks is also locally optimal for each subnetwork. The finiteness of *D*_0_ also ensures, that if *i* and *j* are connected in the original network (*a*_*ij*_ > 0), then they are guaranteed to be connected in a meaningful representation as well, enforcing *b*_*ij*_ > 0, otherwise *D* would diverge. In the opposite case, when we have a connection in the representation, without a corresponding edge in the original graph (*b*_*ij*_ > 0 while *a*_*ij*_ = 0), *b*_*ij*_ does not appear directly in *D*, only globally, as a part of the b_**_ normalization. This density-preserving property leads to a significant computational improvement for sparse networks, since there is no need to build a denser representation matrix, than the input matrix if we keep track of the b_**_ normalization. Nevertheless, the *B* matrix of the optimal representation (where *D* is small) is *close* to *A*, since due to Pinsker's inequality the total variation of the normalized distributions is bounded by *D*^44^


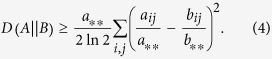


Thus, in the optimal representation of a network all the connected network elements are connected, while having only a strongly suppressed amount of false positive connections. Here we note, that our representation theory can be straightforwardly extended for input networks given by an *H* incidence matrix instead of an adjacency matrix, for details of this case see the Methods section.

### Network visualization and data ordering

Since force-directed layout schemes^28^ have an energy or quality function, optimized by efficient techniques borrowed from many-body physics^45^ and computer science^46^, graph layout could be in principle serve as a quantitative tool. However some of, these popular approaches inherently struggle with an information shortage problem, since the edge weights only provide half the needed data to initialize these techniques. For instance, for the initialization of the widely applied Fruchterman-Reingold^47^ (or for the Kamada-Kawai^48^) method we need to set both the strength of an attractive force (optimal distance) and a repulsive force (spring constant) between the nodes in order to have a balanced system. Due to the lack of sufficient information, such graph layout techniques become somewhat ill-defined and additional subjective considerations are needed to double the information encoded in the input data, traditionally by a nonlinear transformation of the attractive force parameters onto the parameters of the repulsive force^47^. Global optimization techniques, such as information theoretic methods^40^,^41^,^42^,^49^ can, in principle, solve this problem by deriving the needed forces from one single information theoretic quality function.

In strong contrast to usual graph layout schemes, where the nodes are represented by points (without spatial extension) in a *d*-dimensional background space, connected by (straight, curved or more elaborated) lines, in our approach network nodes are extended objects, namely probability distributions (*ρ*(*x*)) over the background space. The *d*-dimensional background space is parametrized by the *d*-dimensional coordinate vector, *x*. Importantly, in our representation the shape of nodes encodes just that additional set of information, which has been lost and then arbitrarily re-introduced in the above mentioned force-directed visualization methods. In the following we consider the simple case of Gaussian distributions – having a width of *σ*, and norm 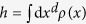, see Eq. (6) of the Methods section –, but we have also tested the non-differentiable case of a homogeneous distribution in a spherical region of radius *σ* leading to similar results. For a given graphical representation the *b*_*ij*_ edge weights are defined as the overlaps of the distributions *ρ*_*i*_ and *ρ*_*j*_, given in the Methods section. For a schematic illustration see Fig. 1b.

The trivial data representation of *B*^0^ can be obtained by an initialization, where all the nodes are at the same position, with the same distribution function (apart from a varying *h*_*i*_ ∝ *a*_i*_ normalization to ensure the proper statistical weight of the nodes). This way, initially *D*_0_ = *I*(*A*) is the mutual information of the input matrix, irrespectively from the chosen distribution function. The numerical optimization can be straightforwardly carried out by a fast and inefficient greedy optimization or a relatively slow, but more efficient simulated annealing scheme starting with an initialization of *B*^0^. As a reasonable compromise, in the differentiable case of Gaussian distributions we use a Newton-Raphson iteration as in the Kamada-Kawai method^48^ (for details see the Supplementary Information), having a run-time of 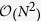 for *N* nodes. Here we note, that similarly to the related t-SNE method^41^,^50^, discussed in the Supplementary Information, the optimization can be in principle carried out in 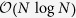 time by applying the Barnes-Hut approximation^45^. 

Independently from the chosen optimization protocol, the finiteness of *D*_0_ ensures that the connected nodes overlap in the layout as well, even for distributions having a finite support. Moreover, independent parts of the network (nodes or sets of nodes without connections between them) tend to be apart from each other in the layout. The density-preserving property of the representation leads to the fact, that even if all the nodes overlap with all other nodes in the layout, the *B* matrix can be kept exactly as sparse as the *A* matrix, while keeping track only of the sum of the b_**_ normalization including the rest of the potential *b*_*ij*_ matrix elements. Additionally, if two rows (and columns) of the input matrix are proportional to each other, then it is optimal to represent them with the same distribution function in the layout, as though the two rows were merged together. 

In the differentiable case, e.g. with Gaussian distributions, our visualization method can be conveniently interpreted as a force-directed method. If the normalized overlap, *b*_*ij*_/b_**_, is smaller at a given edge than the normalized edge weight, *a*_*ij*_/a_**_, then it leads to an attractive force, while the opposite case induces a repulsive force. For details see the Supplementary Information. For Gaussian distributions all nodes overlap in the representations, leading typically to *D* > 0 in the optimal representation. However, for distributions with a finite support, such as the above mentioned homogeneous spheres, perfect layouts with *D* = 0 can be easily achieved even for sparse graphs. In *d* = 2 dimensions this concept is reminiscent to the celebrated concept of planarity^51^. However, our concept can be applied in any dimensions. Furthermore, it goes much beyond planarity, since any network of 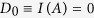 (e.g. a fully connected graph) is perfectly represented in any dimensions by *B*^0^, that is by simply putting all the nodes at the same position.

**Figure 2:**
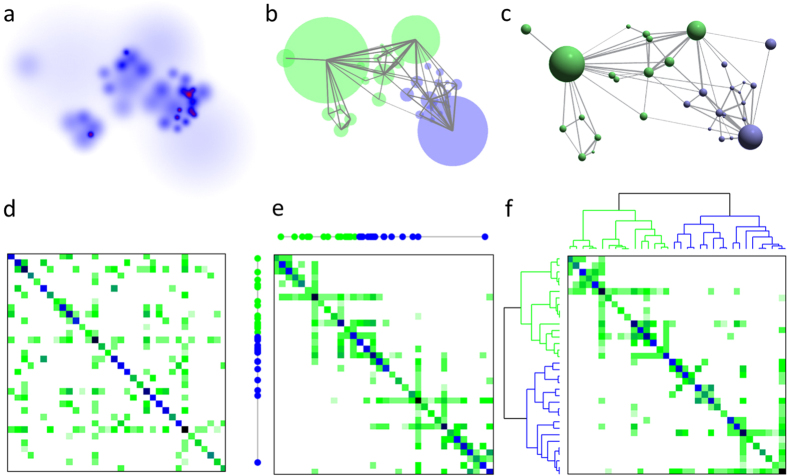
Illustration of the power of our unified representation theory on the Zachary karate club network^52^. The optimal layout (*η* = 2.1%, see Eq. (3)) in terms of *d* = 2 dimensional Gaussians is shown by a density plot in (**a**) and by circles of radiuses *σ*_*i*_ in (**b**). (**c**) the best layout is obtained in *d* = 3 (*η* = 1.7%), where the radiuses of the spheres are chosen to be proportional to *σ*_*i*_. (**d**) the original data matrix of the network with an arbitrary ordering. (**e**) the *d* = 1 layout (*η* = 4.5%) yields an ordering of the original data matrix of the network. (**f**) the optimal coarse-gaining of the data matrix yields a tool to zoom out from the network in accordance with the underlying community structure. The colors indicate our results at the level of two clusters, being equivalent to the ones given by popular community detection techniques, such as the modularity optimization^5^ or the degree-corrected stochastic block model^25^. We note, that the coarse-graining itself does not yield a unique ordering of the nodes, therefore an arbitrarily chosen compatible ordering is shown in this panel.

Our method is illustrated in Fig. 2. on the Zachary karate club network^52^, which became a cornerstone of graph algorithm testing. It is a weighted social network of friendships between *N*_0_ = 34 members of a karate club at a US university, which fell apart after a debate into two communities. While usually the size of the nodes can be chosen arbitrarily, e.g. to illustrate their degree or other relevant characteristics, here the size of the nodes is part of the visualization optimization by reflecting the width of the distribution, indicating relevant information about the layout itself. In fact, the size of a node represents the uncertainty of its position, serving also as a readily available local quality indicator. For illustration of the applicability of our network visualization method to larger collaboration^53^ and information sharing^54^ networks, having more than 10,000 nodes, see the Supplementary Information.

Our network layout technique works in any dimensions, as illustrated in *d* = 1, 2 and 3 in Fig. 2. In each case the communities are clearly recovered and, as expected, the quality of layout becomes better (indicated by a decreasing *η* value) as the dimensionality of the embedding space increases. Nevertheless, the one dimensional case deserves special attention, since it serves as an ordering of the elements as well (after resolving possible degenerations with small perturbations), as illustrated in Fig. 1e.

Since 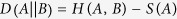, *H*(*A*, *B*) is the (unnormalized) cross-entropy, we can equivalently minimize the cross-entropy for *B*. For a comparison to the known cross-entropy methods^55^,^56^,^57^ see the Supplementary Information. However, as a consequence, the visualization and ordering is perfectly robust against noise in the input matrix elements. This means, that even if the input *A* matrix is just the average of a matrix ensemble, where the *a*_*ij*_ elements have an (arbitrarily) broad distribution, the optimal representation is the same as it were by optimizing for the whole ensemble simultaneously. This extreme robustness follows straightforwardly from the linearity of the *H*(*A*, *B*) cross-entropy in the *a*_*ij*_ matrix elements. Note, however, that the optimal value of the 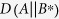 distinguishability is generally shifted by the noise.

When applying a local scheme for the optimization of the representations, we generally run into local minima, in which the layout can not be improved by single node updates, since whole parts of the network should be updated (rescaled, rotated or moved over each other), instead. Being a general difficulty in many optimization problems, it was expected to be insurmountable also in our approach. In the following we show, that the relative entropy based coarse-graining scheme – given in the next section – can, in practice, efficiently help us trough these difficulties in polynomial time.

### Coarse-graining of networks

In the process of coarse-graining we identify groups or clusters of nodes, and try to find the best representation, while averaging out for the details inside the groups. Inside a group, the nodes are replaced by their normalized average, while keeping their degrees fixed. As the simplest example, the coarse-graining of two rows means, that instead of the original *k* and *l* rows, we use two new rows, being proportional to each other, while the *b*_k*_ = *a*_k*_ and *b*_l*_ = *a*_l*_ probabilities are kept fixed


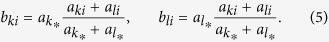


**Figure 3:**
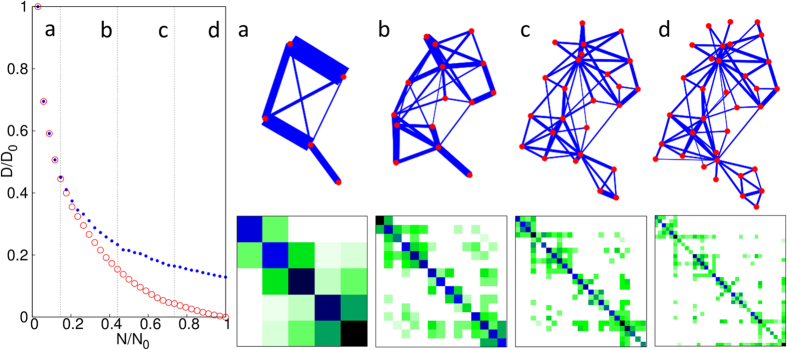
Illustration of our hierarchical visualization technique on the Zachary karate club network^52^. In our hierarchical visualization technique the coarse-graining procedure guides the optimization for the layout in a top-down way. As the *N* number of nodes increases, the relative entropy of both the coarse-grained description (red, ○) and the layout (blue, ●) decreases. The panels (**a**–**d**) show snapshots of the optimal layout and the corresponding coarse-grained input matrix at the level of *N* = 5, 15, 25 and 34 nodes, respectively. For simplicity, here the *h*_*i*_ normalization of each distribution is kept fixed to be ∝ *a*_i*_ during the process, leading finally to *η* = 4.4%.

In other words, we first simply sum up the corresponding rows and obtain a smaller matrix, then inflate this fused matrix back to the original size while keeping the statistical weights of the nodes (degrees) fixed. For an illustration of the smaller, fused data matrices see the lower panels of Fig. 3a–d. For a symmetric adjacency matrix, the coarse-graining step can be also carried out simultaneously and identically for the rows and columns, known as a bi-clustering. The optimal bi-clustering is illustrated in Fig. 2f for the Zachary karate club network. The heights in the shown dendrogram indicate the *D* values of the representations when the fusion step happens.

For the general, technical formulation of our coarse-graining approach and details of the numerical optimization, see the Supplementary Information. As it turns out, for coarse-graining, 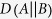 is nothing but the amount of lost mutual information between the rows and columns of the input matrix. In other words, 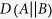 is the amount of lost structural signal during coarse-graining and finally we arrive at a complete loss of structural information, 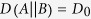. Prevailingly, this final state coincides with the above proposed initialization step of our network layout approach. As a further connection with the graph layout, if two rows (or columns) are proportional to each other, they can be fused together without losing any information, since their coarse-graining leads to no change in the Kullback-Leibler divergence, D.

Since it is generally expected to be an NP-hard problem to find the optimal simplified, coarse-grained description of a network at a given scale, we have to rely on approximate heuristics having a reasonable run-time. In the following we use a local coarse-graining approach, where in each step a pair of rows (and columns) is replaced by coarse-grained ones, giving the best approximative new network in terms of the obtained pairwise *D*-value. This way the optimization can be generally carried out in 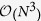 time for *N* nodes. As a common practice, for larger networks we could use the approximation of fusing together a finite amount (eg. 1%) of the nodes in each step instead of a single pair, leading to an improved 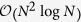 run-time.

As illustrated in Fig. 2f the coarse-graining process creates a hierarchical dendrogram in a bottom-up way, representing the structure of the network at all scales. Here we note, that a direct optimization is also possible for our quality function at a fixed number of groups, creating a clustering. As described in the Supplementary Information in details, our coarse-graining scheme comprises also the case of the overlapping clustering, since it is straightforward to assign a given node to multiple groups as well. As noted there, when considering non-overlapping partitionings with a given number of clusters, our method gives back the degree-corrected stochastic block-model of Karrer and Newman^25^ due to the degree-preservation. Consequently, our coarse-graining approach can be viewed as an overlapping and hierarchical reformulation and generalization of this successful state-of-the-art technique.

### Hierarchical layout

Although the introduced coarse-graining scheme may be of significant interest whenever probabilistic matrices appear, here we focus on its application for network layout, to obtain a hierarchical visualization^58^,^59^,^60^,^61^,^62^,^63^. Our bottom-up coarse-graining results can be readily incorporated into the network layout scheme in a top-down way by initially starting with one node (comprising the whole system), and successively undoing the fusion steps until the original system is recovered. Between each such extension step the layout can be optimized as usual.

**Figure 4:**
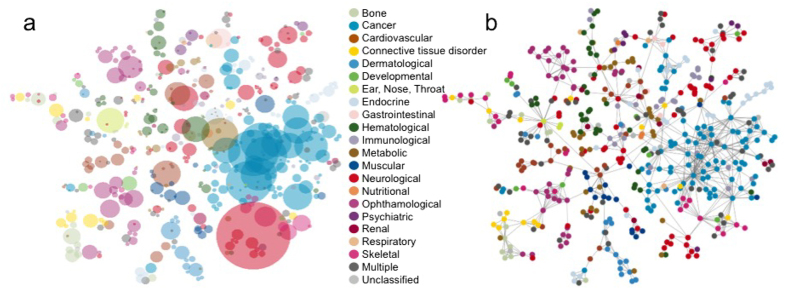
Visualization of the human diseasome. The best obtained layout (*η* = 3.1%) by our hierarchical visualization technique of the human diseasome is shown by circles of radiuses *σ*_*i*_ in (**a**) and by a traditional graph in (**b**). The nodes represent diseases, colored according to known disease categories^64^, while the *σ*_*i*_ width of the distributions in (**a**) indicates the uncertainty of the positions. In the numerical optimization for this network we primarily focused on the positioning of the nodes, thus the optimization for the widths and normalizations was only turned on as a fine-tuning after an initial layout was obtained.

We have found, that this hierarchical layout scheme produces significantly improved layouts – in terms of the final *D* value – compared to a local optimization, such as a simple simulated annealing or Newton-Raphson iteration. By incorporating the coarse-graining in a top-down approach, we first arrange the position of the large-scale parts of the network, and refine the picture in later steps only. The refinement steps happen, when the position and extension of the large-scale parts have already been sufficiently optimized. After such a refinement step, the nodes – moved together so far – are treated separately. At a given scale (having *N* ≤ *N*_0_ nodes), the *D* value of the coarse-graining provides a lower bound for the *D* value of the obtainable layout. Our hierarchical visualization approach is illustrated in Fig. 3. with snapshots of the layout and the coarse-grained representation matrices of the Zachary karate club network^52^ at *N* = 5, 15, 25 and 34. As an illustration on a larger and more challenging network, in Fig. 4. we show the result of the hierarchical visualization on the giant component of the weighted human diseasome network^64^. In this network we have *N*_0_ = 516 nodes, representing diseases, connected by mutually associated genes. The colors indicate the known disease groups, which are found to be well colocalized in the visualization.

## Discussion

In this paper, we have introduced a unified, information theoretic solution for the long-standing problems of matrix ordering, network visualization and data coarse-graining. While establishing a connection between these separated fields for the first time, our unified framework also incorporates some of the known state-of-the art efficient techniques as special cases. In our framework, the steps of the applied algorithms were derived in an *ab inito* way from the same first principles, in strong contrast to the large variety of existing algorithms, lacking such an underlying theory, providing also a clear interpretation of the obtained results.

After establishing the general representation theory, in our paper we first demonstrated that the minimization of relative information yields a novel visualization technique, while representing the *A* input matrix by the *B* co-occurrence matrix of extended distributions, embedded in a *d*-dimensional space. As another application of the same approach, we obtained a hierarchical coarse-graining scheme, when the input matrix is represented by its subsequently coarse-grained versions. Since these applications are two sides of the same representation theory, they turned out to be superbly compatible, leading to an even more powerful hierarchical visualization technique, illustrated on the real-world example of the human diseasome network. Although we have focused on the visualization in *d*-dimensional flat, continuous space, the representation theory can be applied more generally, incorporating also the case of curved or discrete embedding spaces. As a possible future application, we mention the optimal embedding of a (sub)graph into another graph.

We have also shown that our relative entropy-based visualization with e.g. Gaussian node distributions can be naturally interpreted as a force-directed method. Traditional force directed methods prompted huge efforts on the computational side to achieve scalable algorithms applicable for the large data sets in real life. Here we can not and do not wish to compete with such advanced techniques, but we believe that our approach can be a good starting point for further scalable implementations. As a first step towards this goal, we have outlined the possible future directions of computational improvement. Moreover, in the Supplementary Information we illustrated the applicability of our approach on larger scale networks as well. We have also demonstrated, that network visualization is already interesting in one dimension yielding an ordering for the elements of the network. Our efficient coarse-graining scheme can also serve as an unbiased, resolution-limit-free, starting point for the infamously challenging problem of community detection by selecting the best cut of the dendrogram based on appropriately chosen criteria.

Our data representation framework has a broad applicability, starting form either the node-node or edge-edge adjacency matrices or the edge-node incidence matrix of weighted networks, incorporating also the cases of bipartite graphs and hypergraphs. We believe, that our unified representation theory is a powerful tool to gain a deeper understanding of the huge data matrices in science, beyond the limits of existing heuristic algorithms. Since in this paper our primary intention was merely to demonstrate a proof of concept study of our theoretical framework, more detailed analyses of interesting complex networks will be the subject of forthcoming articles.

## Methods

We use the most general form of the input matrices, without assuming their normalization. In accordance, there is no need to normalize the information theoretic measures over 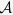, such as the 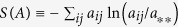 information content or the mutual information between the rows and columns of *A* given by 
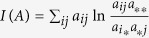
, where 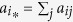 and 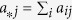. If we start with the *H* edge-node co-occurrence (incidence) matrix instead, suited to describe hypergraphs as well, then *A* ~ *H*^*T*^ *H* is simply given by the elements, 
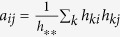
, where 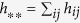. This way we generally have non-zero diagonal entries (*a*_*ii*_). See the Supplementary Information for a discussion on the case with strictly zero diagonals.

The parametrization of the Gaussian distributions used in the visualization is the following in *d*-dimensions


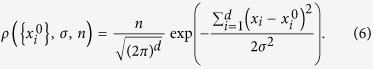


For a given graphical representation the *B* co-occurrence matrix is built up from the overlaps of the distributions *ρ*_*i*_ and *ρ*_*j*_ – analogously to the construction of *A* from *H* – as 
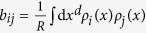
, where 
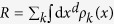
 is an (irrelevant) global normalization factor. Although our network layout works only for symmetric adjacency matrices, the ordering can be extended for hypergraphs with asymmetric *H* matrices as well, since the orderings of the two adjacency matrices *H* *H*^*T*^ and *H*^*T*^ *H* readily yield orderings for both the rows and columns of the matrix, *H*.

For details of the numerical optimization for visualization and coarse-graining see the Supplementary Information. The codes written in C++ using OpenGL are freely available - as command-line programs - upon request.

## Additional Information

**How to cite this article**: Kovács, I. A. *et al.* A unified data representation theory for network visualization, ordering and coarse-graining. *Sci. Rep.* **5**, 13786; doi: 10.1038/srep13786 (2015).

## Supplementary Material

Supplementary Information
