# Supplementary material for: A unified data representation theory for network visualization, ordering and coarse-graining
Source: Sci Rep. 2015 Sep 8;5:13786. doi: 10.1038/srep13786 (PMC4642569; doi:10.1038/srep13786)
Supplement: Supplementary Information [file srep13786-s1.pdf]

Supplementary Information: A unified data representation theory for network visualization, ordering and coarse-graining

István A. Kovács*,[1,2,3] Réka Mizsei,[4] and Péter Csermely[5]

[1] *Wigner Research Centre, Institute for Solid State Physics and Optics, H-1525 Budapest, P.O.Box 49, Hungary*
[2] *Institute of Theoretical Physics, Szeged University, H-6720 Szeged, Hungary*
[3] *Center for Complex Networks Research and Department of Physics, Northeastern University, 110 Forsyth Street, 111 Dana Research Center, Boston, MA 02115, USA*
[4] *Institute of Organic Chemistry, Research Centre for Natural Sciences, Hungarian Academy of Sciences, Pusztaszeri út 59-67, H-1025 Budapest, Hungary*
[5] *Department of Medical Chemistry, Semmelweis University, H-1444 Budapest, P.O.Box 266, Hungary*

(Dated: August 20, 2015)

PACS numbers: 89.75.Fb:Structures and organization in complex systems, 89.75.Hc:Networks and genealogical trees, 89.20.-a:Interdisciplinary applications of physics

Contents

I. Overview of the related methods 2
A. Probabilistic approaches 2
B. Cross-entropy methods for visualization 3
C. Stochastic Neighbor Embedding for visualization 3
D. Stochastic block-models for coarse-graining 4
E. Non-negative matrix factorization 4

II. Numerical optimization for visualization 5
A. Newton-Raphson update in 2 dimensions 5
1. Updating the coordinates 5
2. Updating the widths 6
3. Updating the normalizations 7
B. The case of diagonal elements 7

III. Visualization of larger-scale empirical networks 7
A. Collaboration network: High Energy Physics - Phenomenology 7
B. PGP network 7

IV. Alternative formulation of coarse-graining 7
A. Coarse-graining the rows 8
B. Coarse-graining both the rows and columns 10

V. Greedy optimization for coarse-graining 10
A. Coarse-graining of the rows 11
B. Coarse-graining of both the rows and columns 11
C. Basic notations for coarse-graining 11

References 11

* Electronic address: kovacs.istvan@wigner.mta.hu

I. OVERVIEW OF THE RELATED METHODS

A. Probabilistic approaches

Whenever we apply some random graphs, including benchmarks for clustering, or other generative network models, there is always an underlying probabilistic framework, from which the particular instances are drawn. The common element in these representations is the non-negativity of the matrix elements of the input matrix.

However, when applying information theory on the structure of complex networks, there are different ways of establishing it based on the identification of probability distribution(s) in the given input data, A. In the following we give a brief overview about the possibilities and discuss the existing methods for both visualization and coarse-graining. The possible definitions for the underlying probability distributions are the following.

- Network-level: The full matrix is one probability distribution, thus one system, which can be normalized by $a_{**} = \sum_{ij} a_{ij}$.

- Node-level: Each node, namely each row (or column), in the matrix is a separate probability distribution, which can be normalized independently by $a_{i*} = \sum_j a_{ij}$, $\forall i$. In this case each node is treated independently from the rest of the network.

- Edge-level: Each edge, namely each entry in the matrix, a_{ij}, is a separate probability distribution together with its complementer value $1 - a_{ij}$, normalized independently to 1. In this case each edge is assumed to be independent from the rest of the network. In this case both the adjacency matrix of the existing edges A, and the adjacency matrix of the non-existing edges $1 - A$ is used. In strong contrast to the network-level description, where the probability distribution extends over $N \times N$-points for N nodes, here 2-point distributions are considered of the form $\{a_{ij}, 1 - a_{ij}\}$.

While in this paper we focused on the network-level description, the node and edge level quality functions can be straightforwardly obtained by applying our case for each probability distribution separately. Thus from our network-level quality function

$$Q = D(A||B) = \sum_{ij} a_{ij} \ln \frac{a_{ij} b_{**}}{b_{ij} a_{**}} = \sum_{ij} a_{ij} \ln \frac{a_{ij}}{b_{ij}} + a_{**} \ln \frac{b_{**}}{a_{**}} \tag{1}$$

we arrive at the following node-level quality function

$$Q_N = \sum_i D(A_i||B_i) = \sum_{ij} a_{ij} \ln \frac{a_{ij} b_{i*}}{b_{ij} a_{i*}} = \sum_{ij} a_{ij} \ln \frac{a_{ij}}{b_{ij}} + \sum_i a_{i*} \ln \frac{b_{i*}}{a_{i*}} \tag{2}$$

and edge-level quality function

$$Q_E = \sum_{ij} D(A_{ij}||B_{ij}) = \sum_{ij} a_{ij} \ln \frac{a_{ij}}{b_{ij}} + \sum_{ij} (1 - a_{ij}) \ln \frac{1 - a_{ij}}{1 - b_{ij}} \ . \tag{3}$$

While for visualization the probabilistic approach appears in the literature at all the network [41], node [40] and edge [42, 49] levels, for coarse-graining we are only aware of the network [25, 26] and edge [24, 38] level descriptions. The interesting lack of node-level approaches can be better understood in our framework due to the fact, that for coarse-graining the Q_N quality function gives the same results as the Q quality function for the network-level approach due to the degree preservation in the process.

Here we note, that the network-level probabilistic interpretation usually simply assumes, that the interaction strengths are proportional to (or at least a proxy of) the chance of randomly observing an interaction between the nodes (as applied in our case for the Zachary network [52]), without assuming the independence of the nodes or edges. As for the human diseasome network [62], the edge weights have been directly constructed as co-occurrence values of two diseases over associated genes in perfect accordance to our network-level framework, without any additional assumptions.

In the following we review the most related methods in the literature in somewhat more details to give a clear picture about the similarities and differences.

B. Cross-entropy methods for visualization

Although the minimization of $D(A||B)$ appears in the *minimal discrimination information* approach – also known as the *minimum cross-entropy* (MinxEnt) approach by Kullback [53] –, there the goal is the opposite of ours, namely to find the optimal 'real' distribution, A, while the 'approximate' distribution, B, is kept fixed. In this sense, our optimization is an *inverse* MinxEnt problem [54]. Here we mention, that this kind of inverse optimization appears also as a refinement step to improve the importance sampling in Monte Carlo methods (for highly restricted A-s), under the name of *cross-entropy method* [55].

Here we note, that the concept of cross-entropy have already appeared in the field of graph drawing, such as in the methods of refs. [42, 49]. Besides differences in the final quality function, the most important difference between these methods and ours is, that in our case the relative entropy (or cross entropy) is calculated at the network-level, while these methods operate at the edge-level.

C. Stochastic Neighbor Embedding for visualization

The Stochastic Neighbor Embedding (SNE) [40] is a high-dimensional data visualization tool based on minimizing the Kullback-Leibler divergence between the input and the visual representation. In the case of the traditional SNE, it falls into the category of a node-level probabilistic approach, where the quality function is the sum of the Kullback-Leibler divergences of the rows, representing individual nodes. However, the symmetric SNE [41] works at the network-level, optimizing a single Kullback-Leibler divergence over the whole system.

However, amongst others there are two main differences between these methods and our approach. First, in our method the nodes are represented by extended distribution in the applied (low-dimensional) space, while in SNE and its variants the nodes are represented by points without spatial extensions. As an advantage, in our case the statistical weights of the nodes (the normalization of the distribution) are preserved and proportional to the degrees (a_{i*} row-sums) of the nodes. This way our method is able to adjust to the degrees of the nodes in strong contrast to the SNE-related methods, thus it is generally expected to be more appropriate for real-world networks with highly heterogeneous degree distributions.

Second, in our case the network is represented by the co-occurrence matrix of the probability distributions of the nodes, while in SNE the nodes are represented by a probabilistic matrix, which is obtained by an arbitrary transformation of the distances between the points in the low-dimensional space. Correspondingly, our method not only provides the coordinates of the nodes, but also their probability distribution, representing the uncertainty of the obtained positions. Moreover, in our case the extension of the distributions representing the uncertainty is also an adjustable parameter for each node in contrast to the SNE. As a minor difference, here we also mention, that in the SNE and related methods the diagonal elements are not considered to be parts of the system.

In the t-SNE method [41] the authors claim that it is beneficial to use a heavy-tailed transformation (such as the Student t-distribution) of the distances into probabilities in order to circumvent the observed "crowding" problem. Having namely fixed widths for the distributions it might frequently occur, that we want to put too many neighbors in the vicinity of a given node, while there is simply not enough space for it. In principle, no matter what transformation or distribution we choose for the nodes in the visualization, if the degrees are sufficiently large (as in scale-free networks), we always encounter this problem. This way, the choice of heavy-tailed distributions or transformation rules might generally somewhat reduce the problem, but will not be able to solve it. On the other hand, the crowding problem is trivially solved by adjusting also the spatial extension of the nodes, for any chosen distribution functions, as used in our method. We also note, that in contrast to our general method, the t-SNE approach cannot be applied in $d > 3$ dimensions due to the heavy tail of the Student t-distribution. Nevertheless, it is still an interesting open problem to search for (normalizable) heavy-tailed distributions in our methodology for an even more improved representation of scale-free networks compared to the simple Gaussian case considered above. As mentioned before, our approach also adjusts the statistical weight of each node according to the degree, which again helps to treat the situations with heterogeneous degrees.

However, in the simplest case of Gaussian distributions of equal variance (while only optimizing for the positions) and leaving out the diagonal entries, the formulas for our optimization and for the symmetric SNE coincide when the sum of each row (the degree os each node) is the same, thus in this special case (up to differences in the optimization heuristics) both methods yield the same results.

As an additional significant improvement, in our paper we also presented the hierarchical visualization, being the natural combination of our visualization and coarse-graining. As discussed in the text, the hierarchical approach is expected to produce layouts of much higher quality for large networks, without a significant increase in the computational time. At last, we note, that the t-SNE approach has been significantly improved recently [50] by

applying the Barnes-Hut approximation [45], leading to a computational complexity of $O(N \log N)$ for N nodes, enabling the technique to study networks containing $> 10^7$ nodes. Due to the similarities with the t-SNE the same runtime and system sizes can be reached even in our case in the future enabling the analyses of massively large-scale networks.

D. Stochastic block-models for coarse-graining

Karrer and Newman [25] suggested an improved stochastic block-model (SBM) for the identification of structural patterns in graphs. In this generative model they start from the network-level probabilistic approach and write down the probability of the input matrix assuming that it comes from a given block-model. The improvement comes from the fact that the degrees of the nodes are kept fixed (on average) compared to traditional stochastic block-models, leading to much less powerful results.

In the degree-corrected SBM, when looking for partitions of the input matrix with a given number of clusters, the maximum probability (likelihood) solution coincides to the one having the minimal Kullback-Leibler divergence, $D(A||B)$, exactly as in our case. Recently it turned out [26] that for bipartite graphs it leads to higher quality results, if the bipartite structure is *a priori* enforced, instead of letting the method to learn it during the optimization.

Since our coarse-graining approach is formulated for the more general case including overlapping partitions, the state-of-the-art degree-corrected SBM can be viewed as a special case of our general coarse-graining method. As a practical difference, in our case we apply the coarse-graining hierarchically, not only at a given number of clusters. We note, however, that the hierarchical approach at a fixed number of clusters gives in general slightly different results from the direct optimization at the same number of clusters. Computationally this is not surprising, since the hierarchical approach has a polynomial run-time, while the clustering problem is known to be NP-hard. Based on this observation one might think, that the direct clustering is superior to the hierarchical approach, which is true in quality (D value), but not entirely true in purpose. The reason is, that the aim in the hierarchical coarse-graining is to find a dendrogram, or global hierarchical description, which is as optimal as possible at *all* scales of the network, not only at a fixed scale. This way the obtained dendrogram might not be optimal at all individual scales, while providing a much more detailed, global representation about the network.

Here we mention, that in the mathematical literature concentrated around graph-limits and graphons [38] and in the case of benchmark graphs for clustering [24], the term SBM has a different meaning. Namely, in these works, the SBM is formulated in the edge-level probabilistic framework, assuming the independence of all the edges from each other, in strong contrast to the network-level description.

E. Non-negative matrix factorization

While in the case of coarse-graining the aim is to fuse together parts of the input matrix, in the powerful technique of Non-negative matrix factorization [32] the aim is to divide the existing parts into subunits, which appear to be together more often. This way the goal is to find the most relevant building blocks. Given the number of blocks to use, r, the NMF uses a quality function, reminiscent to the $D(A||B)$ Kullback-Leibler divergence in our studies

$$F = -D(A||B) + a_{**} \ln b_{**} - b_{**} \ . \tag{4}$$

Here the first term is independent of b_{**} and the second term only fixes the value of b_{**} to be equal to a_{**}, thus the optimization is practically equivalent to the minimization of $D(A||B)$. As in our case A (and B) can be a non-symmetric matrix of size $N \times r$. The main difference lies in the choice of the representation B, which is chosen to be $B = WH$, where both W $(N \times r)$ and H $(r \times M)$ are to be found, having non-negative matrix elements, with a fixed number of building blocks, r. The idea is to model the input matrix, A as a linear combination (W) of appropriate hidden variables, H. Although this is a hard simultaneous optimization problem for W and H, for practical applications there is an efficient iterative approach to find locally optimal solutions, starting from random initial conditions [32].

II. NUMERICAL OPTIMIZATION FOR VISUALIZATION

For the numerical optimization of the network layout, we have implemented a simple, general purpose simulated annealing scheme. For Gaussian distributions we have also worked out a much faster Newton-Raphson update, which has been also applied in the Kamada-Kawai method. In practice, we used a separate Newton-Raphson iteration step for the d coordinates of the nodes in d spatial dimensions and for the σ_i widths and h_i normalizations of the distributions.

In each iteration step of the Newton-Raphson method, the node with the largest gradient amplitude ($||J||$) was updated in the direction and with a parameter step size, obtained by the second derivative matrix, $\mathcal{F}$ as $-\mathcal{F}^{-1}J$. Since $\mathcal{F}$ is not always positive definite, special care was needed when the relative entropy increased in such a step. In such a case, a sufficiently small step size was applied in the direction of the gradient vector, instead. This way our technique has the same computational complexity as the widely applied Kamada-Kawai method (after the initial calculation of pairwise graph-theoretic distances).

While the original, input matrix is given by A, the visualization generates the matrix of the pairwise overlaps of the node distributions, marked as B. In our approach we minimize the relative entropy between the two distributions, $D(A||B)$, which measures the extra description length, when B is used to encode the data described by A,

$$D(A||B) = \sum_{ij} a_{ij} \ln \frac{a_{ij} b_{**}}{b_{ij} a_{**}} \geq 0 \, . \tag{5}$$

Here an asterisks indicates and index for which we summed up. During optimization the a_{ij} matrix elements of the A input matrix were kept fixed, while the values of b_{ij} changed due to the variation of the x_i, y_i, σ_i (and h_i) parameters of the d-dimensional Gaussian distributions of the nodes, given by

$$\rho(\{x_i^0\}, \sigma, n) = \frac{n}{\sqrt{(2\pi)^d}} \exp\left(-\frac{\sum_{i=1}^{d}(x_i - x_i^0)^2}{2\sigma^2}\right) . \tag{6}$$

The overlap matrix elements of the node distributions in $d = 2$ dimensions, with notations x and y for the two coordinates, were given by

$$b_{ij} = \frac{h_i h_j}{2\pi(\sigma_i^2 + \sigma_j^2)} \exp\left(-\frac{(x_i - x_j)^2 + (y_i - y_j)^2}{2(\sigma_i^2 + \sigma_j^2)}\right) . \tag{7}$$

A. Newton-Raphson update in 2 dimensions

For a Newton-Raphson iteration step we need to calculate the first and second derivatives of the D relative entropy as the function of the parameters of each node.

1. *Updating the coordinates*

When differentiating according to the coordinates, we obtain

$$\frac{\partial b_{kj}}{\partial x_k} = -\frac{x_k - x_j}{\sigma_k^2 + \sigma_j^2} b_{kj} \, , \tag{8}$$

$$\frac{\partial D}{\partial x_k} = -2a_{**} \sum_j \frac{x_k - x_j}{\sigma_k^2 + \sigma_j^2} \left(\frac{b_{kj}}{b_{**}} - \frac{a_{kj}}{a_{**}}\right) . \tag{9}$$

From this we can see, that $\frac{b_{kj}}{b_{**}} > \frac{a_{kj}}{a_{**}}$ induces a repulsive force, while the opposite case leads to an attractive force. In order to have an efficient numerical implementation we introduce the following variables.

$$\alpha_{kj}^x = -2a_{**} \frac{x_k - x_j}{\sigma_k^2 + \sigma_j^2} b_{kj} \, , \tag{10}$$

$$\beta_{kj}^x = -2 \frac{x_k - x_j}{\sigma_k^2 + \sigma_j^2} a_{kj} \, . \tag{11}$$

Here the superscript x indicates, that we now consider the x direction in the formulas. This way the J gradient vector has the following x-component

$$j_x \equiv \frac{\partial D}{\partial x_k} = \frac{\alpha^x_{k*}}{b_{**}} - \beta^x_{k*} \ . \tag{12}$$

Consequently, while using α^x and β^x, $\frac{\partial D}{\partial x_k}$ can be calculated in $\mathcal{O}(N)$ time $\forall k$, instead of the $\mathcal{O}(N^2)$ approach of a direct evaluation. For the y direction the same formulas apply with the substitution, $x \leftrightarrow y$.

During the Newton-Raphson method that node, k, was updated, for which $||J|| \equiv j_x^2 + j_y^2$ was the largest. The $\mathcal{F}$ second derivative matrix had the following elements at a given node, k.

$$\begin{aligned} f_{xx} \equiv \frac{\partial^2 D}{\partial x_k^2} = -2\frac{a_{**}}{b_{**}} \sum_j \frac{b_{kj}}{\sigma_k^2 + \sigma_j^2} + 2\sum_j \frac{a_{kj}}{\sigma_k^2+\sigma_j^2} \\ -\frac{(\alpha^x_{k*})^2}{2a_{**}b_{**}^2} - \frac{1}{b_{**}} \sum_j \frac{x_k - x_j}{\sigma_k^2+\sigma_j^2}\alpha^x_{kj} \ . \end{aligned} \tag{13}$$

$$f_{xy} \equiv \frac{\partial^2 D}{\partial x_k \partial y_k} = -2\frac{\alpha^x_{k*}\alpha^y_{k*}}{2a_{**}b_{**}^2} - \frac{1}{b_{**}} \sum_j \frac{y_k - y_j}{\sigma_k^2+\sigma_j^2}\alpha^x_{kj} \ . \tag{14}$$

In the Newton-Raphson method the step size in the x and y directions were automatically given by the vector $-\mathcal{F}^{-1}J$, if $\Delta \equiv f_{xx}f_{yy} - f_{xy}^2 \neq 0$. As a result, in the x- and y-directions we obtained

$$\delta_x = \frac{1}{\Delta}(f_{xy}j_y - f_{yy}j_x), \ \delta_y = \frac{1}{\Delta}(f_{xy}j_x - f_{xx}j_y) \ . \tag{15}$$

Since $\mathcal{F}$ is not always positive definite (not even in the traditional Kamada-Kawai method), special care was needed when the relative entropy increased in such a step. In such a case, a sufficiently small step size was applied in the direction of the gradient vector, instead of the direction given by $\mathcal{F}$. In our implementation we started with the same step size as before and iteratively kept dividing it by two, until the relative entropy decreased.

2. Updating the widths

The widths were updated separately in a similar manner (there was only one variable at each node). In order to have an efficient implementation, we first introduced the following variable

$$\gamma_{kj} = \frac{\sigma_k}{\sigma_k^2+\sigma_j^2}\left(\frac{(x_k-x_j)^2+(y_k-y_j)^2}{\sigma_k^2+\sigma_j^2} - 2\right) \ , \tag{16}$$

with which

$$\frac{\partial b_{kj}}{\partial \sigma_k} = b_{kj}\gamma_{kj} \ . \tag{17}$$

$$\frac{\partial D}{\partial \sigma_k} = -2\sum_j a_{kj}\gamma_{kj} + 2\frac{a_{**}}{b_{**}}\sum_j b_{kj}\gamma_{kj} \ . \tag{18}$$

The second derivative at a given node k was

$$\begin{aligned} \frac{\partial^2 D}{\partial \sigma_k^2} = -2\sum_j \frac{a_{kj}}{\sigma_k^2+\sigma_j^2}\epsilon_{kj} - 2\frac{a_{**}}{b_{**}}\sum_j \frac{b_{kj}}{\sigma_k^2+\sigma_j^2}\epsilon_{kj} \\ -\frac{4a_{**}}{b_{**}^2}\left(\sum_j b_{kj}\gamma_{kj}\right)^2 + \frac{2a_{**}}{b_{**}}\sum_j b_{kj}\gamma_{kj}^2 \ , \end{aligned} \tag{19}$$

where we used the notation

$$\epsilon_{kj} = \gamma_{kj}\frac{\sigma_j^2 - 2\sigma_k^2}{\sigma_k} - \frac{2\sigma_k^2}{\sigma_k^2+\sigma_j^2} \ . \tag{20}$$

3. Updating the normalizations

Although in many applications it is more natural to keep the normalizations fixed at their original value, it generally leads to improved representations if we update the h_i normalization values as well during the optimization, so we provide here the details for these steps.

$$\frac{\partial b_{kj}}{\partial h_k} = \frac{b_{kj}}{h_k}, \tag{21}$$

$$\frac{\partial D}{\partial h_k} = \frac{1}{b_{**}}\frac{2a_{**}b_{k*}}{h_k} - \frac{2a_{k*}}{h_k}. \tag{22}$$

The second derivative at a given node k was

$$\frac{\partial^2 D}{\partial h_k^2} = -\frac{4a_{**}b_{k*}^2}{h_k^2 b_{**}^2} + \frac{2a_{k*}}{h_k^2}. \tag{23}$$

B. The case of diagonal elements

The above formulas hold for the diagonal b_{ii} self-overlap elements as well. However, the b_{ii} values do not change during repositioning the nodes, but only by updating the σ_i widths or h_i normalizations of the Gaussians. Nevertheless, in practice, special care may be needed for the diagonal elements, describing the probability of the co-occurrence of an element with itself. If the nodes represent individual entities in A, rather than some properties or groups, then such self co-occurrences are impossible leading to $a_{ii} \equiv 0$, which can be included in the representation scheme as well, by requiring $b_{ii} \equiv 0$. While the solution of this case is rather straightforward, for the sake of simplicity we omitted its detailed study.

III. VISUALIZATION OF LARGER-SCALE EMPIRICAL NETWORKS

In this section we illustrate the applicability of our visualization approach for some empirical networks containing more than $10,000$ nodes, namely the high energy physics collaboration network [63] and the secure information sharing over a PGP network [64]. In both cases we consider the largest connected components and provide the number of nodes and edges accordingly. For simplicity, here we use the Newton-Raphson update for the positions only.

A. Collaboration network: High Energy Physics - Phenomenology

This undirected, unweighted network containing $N = 11,204$ nodes and $E = 117,649$ edges covers the collaboration between authors of manuscripts submitted to the HEP-PH (High Energy Physics - Phenomenology) section of the arXiv e-print server from January 1993 to April 2003 (124 months) [63]. Two authors are connected, if they published together a paper.

B. PGP network

This undirected, unweighted network shows the secure information interchange through the PGP (Pretty Good Privacy) algorithm of $N = 10,680$ users as nodes nodes, leading to $E = 24,316$ edges [64].

IV. ALTERNATIVE FORMULATION OF COARSE-GRAINING

In this section we show a simple, intuitive interpretation of our coarse-graining approach.

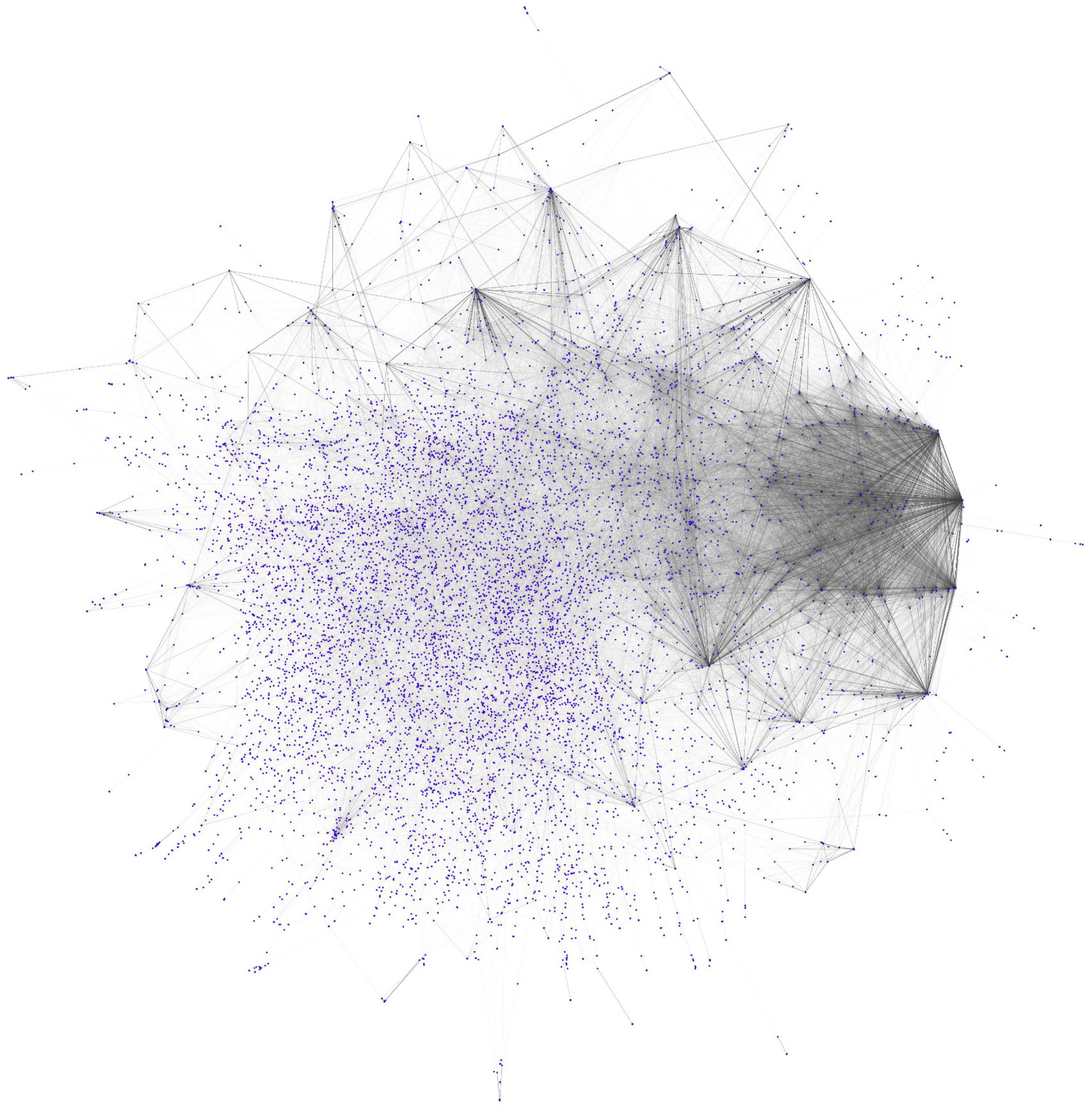

Supplementary Fig. 1: **Collaboration network.** Visualization of the collaboration in the "High Energy Physics - Phenomenology" section of the arXiv pre-print server from January 1993 to April 2003, containing $N = 11,204$ nodes and $E = 117,649$ edges.

A. Coarse-graining the rows

A grouping or coarse-graining, M, of the rows of the input matrix A can be generally described by the fusion matrix U as

$$m_{ij} = \sum_k u_{ik} a_{kj} \ , \tag{24}$$

where $u_{*k} = 1$, $\forall k$. Instead of this reduced size matrix, in our coarse-graining we used a (practically equivalent), averaged out representation, B, of the original size given by the elements

$$b_{ij} = a_{i*} \sum_k u_{ki} \frac{m_{kj}}{m_{k*}} \ . \tag{25}$$

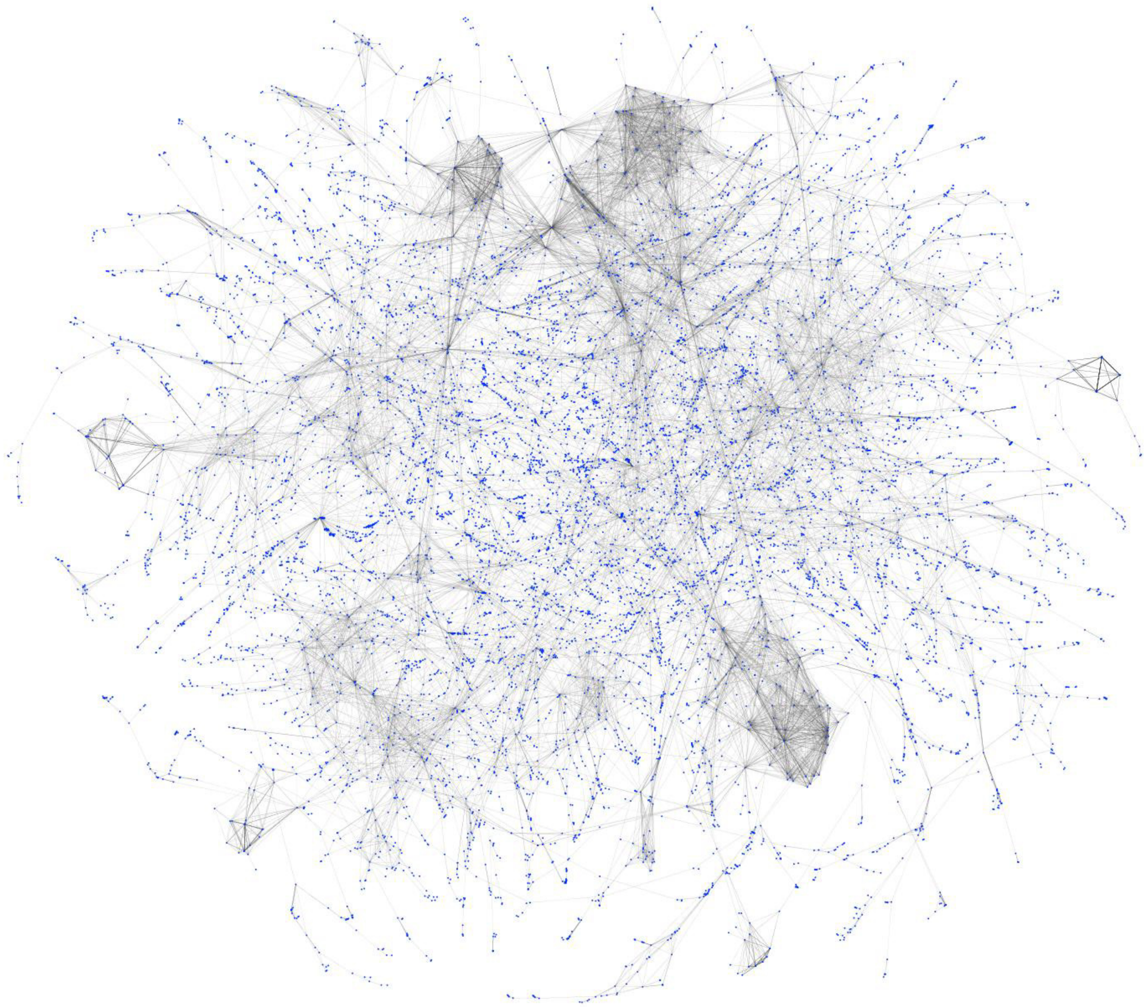

Supplementary Fig. 2: **PGP network of information sharing.** This undirected, unweighted network contains $N = 10,680$ nodes and $E = 24,316$ edges, connected in a complex, heterogeneous way, as unveiled by our visualization.

By considering partitionings of the rows, without overlaps, each row i had a unique label $\sigma(i)$ yielding its cluster. With this notation

$$b_{ij} = a_{i*} \frac{m_{\sigma(i)j}}{m_{\sigma(i)*}} \ . \tag{26}$$

By substituting this into Eq. (5), and changing the indices $\sigma(i) \rightarrow i$ we arrive at

$$D = \sum_{ij} a_{ij} \ln \frac{a_{ij}}{a_{i*}} - \sum_{ij} m_{ij} \ln \frac{m_{ij}}{m_{i*}} \ , \tag{27}$$

which is simply

$$D = I(R, C) - I(r, C) \ , \tag{28}$$

where r means the coarse-grained set of rows, R. Since the $I(R, C) \equiv D_0$ mutual information can be interpreted as the amount of structural 'signal' in the original data, D is the amount of lost structural signal during coarse-graining.

B. Coarse-graining both the rows and columns

The simultaneous coarse-graining of the rows and columns of A was given by the matrix elements

$$w_{ij} = \sum_k u_{ik} v_{jl} a_{kl} \ , \tag{29}$$

where $v_{*l} = 1$, $\forall l$. The averaged out representation, B, of the original size was given in this case by the elements

$$b_{ij} = a_{i*} a_{*j} \sum_{kl} u_{ki} v_{lj} \frac{w_{kl}}{w_{k*} w_{*l}} \ . \tag{30}$$

By considering partitionings, this can be written in the form of

$$b_{ij} = a_{i*} a_{*j} \frac{w_{\sigma(k)\sigma(l)}}{w_{\sigma(k)*} w_{*\sigma(l)}} \ . \tag{31}$$

By substituting this into Eq. (5), and changing the indices as before, we arrive at

$$D = \sum_{ij} a_{ij} \ln \frac{a_{ij}}{a_{i*} a_{*j}} - \sum_{ij} w_{ij} \ln \frac{w_{ij}}{w_{i*} w_{*j}} \ , \tag{32}$$

which is simply

$$D = I(R, C) - I(r, c) \ , \tag{33}$$

where r (c) means the coarse-grained R (C). Thus, it is true also in this case, that D can be interpreted as the amount of lost structural signal during coarse-graining. For a graphical representation of these considerations see Fig. 1. of the Supplementary Information.

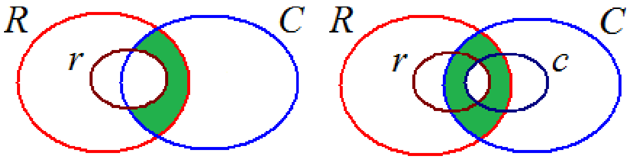

Supplementary Fig. 3: **Graphical representation of the relative entropy, D, used in the hierarchical coarse-graining.** In the information content diagram the shaded (green) area indicates D, which is found to be the amount of the lost mutual information between the rows (R) and the columns (C). The coarse-grained rows and columns are denoted by r and c, respectively. Left: coarse-graining for the rows (R) only. Right: simultaneous coarse-graining of the rows and columns.

V. GREEDY OPTIMIZATION FOR COARSE-GRAINING

Since in a coarse-graining step the B representation matrix is modified, for the remaining steps the D difference should be updated if at least one member of the pair is neighbor of the fused elements. Although it seems to be tedious in a later step to measure D always from the original input matrix for a given (k, l) pair, there is a simple way to calculate this from the actually existing coarse-grained data alone. If D_k and D_l are the values when the rows (and columns) k and l were formed via fusion (being zero initially), then from the apparent D' value – measuring the formation of a bond directly from the coarse-grained rows k and l – we got

$$D = D_k + D_l + D' \ . \tag{34}$$

This results is valid both for the coarse-graining of the rows and for the simultaneous coarse-graining of both the rows and columns.

A. Coarse-graining of the rows

In the following we summarize the numerical details of coarse-graining the rows of a matrix with N_r rows and N_c columns. For each pair of rows the δ difference of the D relative entropy value for the fusion step could be calculated independently from the other pairs. Thus after a fusion step only the δ values of the new row with the rest of the rows were needed to be calculated in $\mathcal{O}(N_c)$ time. Since in each step the pair with the lowest δ value was fused, we needed to select the lowest value before each step, which could be conveniently done with a binary heap data structure in $\mathcal{O}(\ln N_r)$ time. Altogether we finished in $\mathcal{O}\left(N_r^2 N_c\right)$ time.

B. Coarse-graining of both the rows and columns

In the following we overview the numerical details of the simultaneous coarse-graining of both the rows and columns of a symmetric matrix with N rows and columns. In this case the fusion of two node pairs is generally not independent, thus besides calculating the δ values of the new row, all the other values may be needed to be updated. Fortunately, this can be done in constant time between rows i and j. After the fusion of rows a and b, δ_{ij} must be increased by $2\Delta_{ij}$, where

$$\begin{aligned}\Delta_{ij} &= -w_{ia}\ln w_{ia} - w_{ja}\ln w_{ja} - w_{ib}\ln w_{ib} - w_{jb}\ln w_{jb} \\ &+(w_{ia}+w_{ja})\ln(w_{ia}+w_{ja}) + (w_{ib}+w_{jb})\ln(w_{ib}+w_{jb}) \\ &+(w_{ia}+w_{ib})\ln(w_{ia}+w_{ib}) + (w_{ja}+w_{jb})\ln(w_{ja}+w_{jb}) \\ &-(w_{ia}+w_{ja}+w_{ib}+w_{jb})\ln(w_{ia}+w_{ja}+w_{ib}+w_{jb})\ .\end{aligned} \tag{35}$$

Altogether the whole process took $\mathcal{O}(N^3)$ time.

C. Basic notations for coarse-graining

With the notations $m_{ij} = \sum_k u_{ik}a_{kj}$, $n_{ij} = \sum_k a_{ik}v_{jk}$ and $w_{ij} = \sum_{kl} u_{ik}a_{kl}v_{jl}$ the relevant entropy measures can be expressed as follows.

$$S(R) = -\sum_i a_{i*}\ln\frac{a_{i*}}{a_{**}},\ S(C) = -\sum_i a_{*i}\ln\frac{a_{*i}}{a_{**}} \tag{36}$$

$$S(r) = -\sum_i m_{i*}\ln\frac{m_{i*}}{a_{**}}\,; S(c) = -\sum_i n_{*i}\ln\frac{n_{*i}}{a_{**}} \tag{37}$$

$$S(r,C) = -\sum_{ij} m_{ij}\ln\frac{m_{ij}}{a_{**}},\ S(R,c) = -\sum_{ij} n_{ij}\ln\frac{n_{ij}}{a_{**}} \tag{38}$$

$$S(R,C) = -\sum_{ij} a_{ij}\ln\frac{a_{ij}}{a_{**}},\ S(r,c) = -\sum_{ij} w_{ij}\ln\frac{w_{ij}}{a_{**}} \tag{39}$$

From these we could deduce the used measures of mutual information for any X and Y as $I(X,Y) = S(X) + S(Y) - S(X,Y)$.

---

[1] Newman, M. E. J. Networks: An Introduction. (Oxford Univ. Press, 2010).
[2] Albert, R. & Barabási, A.-L. Statistical mechanics of complex networks. *Reviews of Modern Physics* **74,** 47-97 (2002).
[3] Newman, M. E. J. Assortative mixing in networks. *Phys. Rev. Lett.* **89,** 208701 (2002).
[4] Reshef, D. N. et al. Detecting novel associations in large data sets. *Science* **334,** 1518-1524 (2011).
[5] Girvan, M. & Newman, M. E. J. Community structure in social and biological networks. *Proc. Natl Acad. Sci. USA* **99,** 7821-7826 (2002).
[6] Newman, M. E. J. Communities, modules and large-scale structure in networks. *Nature Physics* **8,** 25-31 (2012).

[7] Fortunato, S. Community detection in graphs. *Phys. Rep.* **486,** 75-174 (2010).
[8] Kovács, I. A., Palotai, R., Szalay, M. S. & Csermely, P. Community landscapes: an integrative approach to determine overlapping network module hierarchy, identify key nodes and predict network dynamics. *PLoS ONE* **5,** e12528 (2010).
[9] Olhede, S. C. & Wolfe, P. J. Network histograms and universality of blockmodel approximation. *Proc. Natl Acad. Sci. USA* **111,** 14722-14727 (2014).
[10] Bickel P. J., Chen A. A nonparametric view of network models and Newman-Girvan and other modularities. *Proc. Natl. Acad. Sci. USA* **106** (50):2106821073. (2009).
[11] Bickel P. J., Sarkar P. Hypothesis testing for automated community detection in networks. arXiv:1311.2694. (2013) (Date of access:15/02/2015).
[12] King, I. P. An automatic reordering scheme for simultaneous equations derived from network analysis. *Int. J. Numer. Methods*, **2,** 523-533 (1970).
[13] George A. & Liu, J. W.-H. Computer solution of large sparse positive definite systems. (Prentice-Hall Inc, 1981).
[14] West, D. B. Introduction to graph theory 2nd edn. (Prentice-Hall Inc, 2001).
[15] Song, C., Havlin, S. & Makse, H. A. Self-similarity of complex networks. *Nature* **433,** 392-395 (2005).
[16] Gfeller, D. & De Los Rios, P. Spectral coarse graining of complex networks. *Phys. Rev. Lett.* **99,** 038701 (2007).
[17] Sales-Pardo, M., Guimera, R., Moreira, A. A. & Amaral L. A. N. Extracting the hierarchical organization of complex systems. *Proc. Natl. Acad. Sci. USA* **104,** 15224-15229 (2007).
[18] Ravasz, E., Somera, A. L., Mongru, D. A., Oltvai, Z. N. & Barabási, A.-L. Hierarchical organization of modularity in metabolic networks. *Science* **297,** 1551-1555 (2002).
[19] Radicchi, F., Ramasco, J. J., Barrat, A. & Fortunato, S. Complex networks renormalization: flows and fixed points. *Phys. Rev. Lett.* **101,** 148701 (2008).
[20] Rozenfeld, H. D., Song, C. & A. Makse, H. A. Small-world to fractal transition in complex networks: a renormalization group approach. *Phys. Rev. Lett.* **104,** 025701 (2010).
[21] Walshaw, C. A multilevel approach to the travelling salesman problem. *Oper. Res.*, **50,** 862-877 (2002).
[22] Walshaw, C. Multilevel refinement for combinatorial optimisation problems. *Annals of Operations Research* **131,** 325-372 (2004).
[23] Ahn, Y.-Y., Bagrow J. P. & Lehmann S. Link communities reveal multiscale complexity in networks *Nature* **1038**, 1-5 (2010).
[24] Lancichinetti, A., Fortunate, S. & Radicchi, F., Benchmark graphs for testing community detection algorithms, *Phys. Rev. E* **78,** 046110 (2008).
[25] Karrer, B. & Newman, M. E. J., Stochastic blockmodels and community structure in networks. *Phys. Rev. E* **83,** 016107 (2011).
[26] Larremore, D. B., Clauset, A. & Jacobs, A. Z., Efficiently inferring community structure in bipartite networks. *Phys. Rev. E* **90,** 012805 (2014).
[27] Di Battista, G., Eades, P., Tamassia, R. & Tollis, I.G. Graph Drawing: Algorithms for the Visualization of Graphs. (Prentice-Hall Inc, 1998).
[28] Kobourov, S. G. Spring embedders and force-directed graph drawing algorithms. arXiv:1201.3011 (2012) (Date of access:15/02/2015).
[29] Graph Drawing, Symposium on Graph Drawing GD'96 (ed North, S.), (Springer-Verlag, Berlin, 1997).
[30] Garey, M. R. & Johnson, D. S. Computers and Intractability: A Guide to the Theory of NP-Completeness. (W.H. Freeman and Co., 1979).
[31] Kinney, J. B. & Atwal, G. S. Equitability, mutual information, and the maximal information coefficient. *Proc. Natl. Acad. Sci. USA* **111,** 3354-3359 (2014).
[32] Lee, D. D., &Seung, H. S., Learning the parts of objects by non-negative matrix factorization. *Nature* **401,** 788-791 (1999).
[33] Slonim, N., Atwal, G. S., Tkačik, G. & Bialek, W. Information-based clustering. *Proc. Natl. Acad. Sci. USA* **102,** 18297-18302 (2005).
[34] Rosvall, M. & Bergstrom, C. T. An information-theoretic framework for resolving community structure in complex networks. *Proc. Natl. Acad. Sci. USA* **104,** 7327-7331 (2007).
[35] Rosvall, M., Axelsson, D. & Bergstrom, C. T. The map equation. *Eur. Phys. J. Special Topics* **178,** 13 -23 (2009).
[36] Zanin, M., Sousa, P. A. & Menasalvas, E. Information content: assessing meso-scale structures in complex networks. *Europhys. Lett.* **106,** 30001 (2014).
[37] Allen, B., Stacey, B. C. & Bar-Yam, Y. An information-theoretic formalism for multiscale structure in complex systems. arXiv:1409.4708 (2014) (Date of access:15/02/2015).
[38] Lovász, L., Large networks and graph limits, volume 60 of American Mathematical Society Colloquium Publications. American Mathematical Society, Providence, RI, (2012).
[39] Kullback, S. & Leibler, R. A. On information and sufficiency. *Annals of Mathematical Statistics* **22,** 79-86 (1951).
[40] Hinton, G., & Roweis, S., Stochastic Neighbor Embedding, in Advances in Neural Information Processing Systems, Vol. 15, 833-840 (The MIT Press, Cambridge, 2002).
[41] van der Maaten, L., & Hinton, G., Visualizing Data using t-SNE, *Journal of Machine Learning Research*, **9,** 2579-2605 (2008).
[42] Yamada, T., Saito, K. & Ueda, N. Cross-entropy directed embedding of network data, *Proceedings of the 20th International Conference on Machine Learning (ICML2003)*, 832-839 (2003).
[43] Grünwald, P. D., The Minimum Description Length Principle, (MIT Press, 2007).
[44] See, e.g., Cover, Th. M. & Thomas, J. A. Elements of Information Theory 1st edn, Lemma 12.6.1, 300-301 (John Wiley & Sons, 1991).
[45] Barnes, J. & Hut, P. A hierarchical O(NlogN) force-calculation algorithm. *Nature*, **324,** 446-449 (1986).
[46] Gansner, E. R., Koren, Y. & North, S. in Graph drawing by stress majorization, Vol. 3383 (ed Pach J.), 239-250 (Springer-Verlag, 2004).
[47] Fruchterman, T. M. & Reingold, E. M. Graph Drawing by Force-Directed Placement, *Software: Practice & Experience* **21,** 1129-1164 (1991).
[48] Kamada, T. & Kawai, S. An algorithm for drawing general undirected graphs. *Information Processing Letters* (Elsevier) **31,** 7-15 (1989).
[49] Estévez, P. A., Figueroa, C. J. & Saito, K. Cross-entropy embedding of high-dimensional data using the neural gas model. *Neural Networks*, **18,** 727-737 (2005).
[50] van der Maaten, L. J. P., Accelerating t-SNE using Tree-Based Algorithms. *Journal of Machine Learning Research* **15** 3221-3245 (2014).

[51] Hopcroft, J. & Tarjan, R. E. Efficient planarity testing. *Journal of the Association for Computing Machinery* **21,** 549-568 (1974).

[52] Zachary, W. W. An information flow model for conflict and fission in small groups. *Journal of Anthropological Research* **33,** 452-473 (1977).

[53] Kullback, S. Information Theory and Statistics, (John Wiley: New York, NY, USA, 1959).

[54] Kapur, J. N. & Kesavan, H. K., The inverse MaxEnt and MinxEnt principles and their applications, in Maximum Entropy and Bayesian Methods, Fundamental Theories in Physics, Springer Netherlands, **39,** 433-450 (1990).

[55] Rubinstein, R. Y., The cross-entropy method for combinatorial and continuous optimization. *Method. Comput. Appl. Probab.* **1,** 127-190 (1999).

[56] Gajer, P., Goodrich, M. T. & Kobourov, S. G. A multi-dimensional approach to force-directed layouts of large graphs, *Computational Geometry: Theory and Applications* **29,** 3-18 (2004).

[57] Harel, D. & Koren, Y. A fast multi-scale method for drawing large graphs. *J. Graph Algorithms and Applications*, **6,** 179-202 (2002).

[58] Walshaw, C. A multilevel algorithm for force-directed graph drawing. *J. Graph Algorithms Appl.*, **7,** 253-285 (2003).

[59] Hu, Y. F. Efficient and high quality force-directed graph drawing. *The Mathematica Journal*, **10,** 37-71 (2006).

[60] Szalay-Bekő, M., Palotai, R., Szappanos, B., Kovács, I. A., Papp, B. & Csermely P., ModuLand plug-in for Cytoscape: determination of hierarchical layers of overlapping network modules and community centrality. *Bioinformatics* **28,** 2202-2204 (2012).

[61] Six, J. M., & Tollis, I. G. in Software Visualization, Vol. 734, (ed Zhang, K.) Ch. 14, 413-437 (Springer US, 2003).

[62] Goh, K.-I. et al. The human disease network. *Proc. Natl. Acad. Sci. USA* **104,** 8685-8690 (2007).
Goh, K.-I., Cusick, M., Valle, D., Childs, B., Vidal, M. & Barabási, A.-L., The human disease network (the human diseasome)., (2006) (Date of access:15/02/2015)
`http://www.barabasilab.com/pubs/CCNR-ALB_Publications/200705-14_PNAS-HumanDisease/Suppl/index.htm`

[63] Leskovec, J., Kleinberg, J. & Faloutsos, C., Graph Evolution: Densification and Shrinking Diameters. ACM Transactions on Knowledge Discovery from Data (ACM TKDD), 1(1), (2007). Data is available at: `http://snap.stanford.edu/data/ca-HepPh.html`

[64] Boguña, M., Pastor-Satorras, R., Diaz-Guilera, A., & Arenas, A., Models of social networks based on social distance attachment. *Phys. Rev. E*, **70,** 056122 (2004). Data is available at: `http://deim.urv.cat/ alexandre.arenas/data/welcome.htm`
